# Correlation of the severity of anemia in patients undergoing total joint arthroplasty with preoperative deep vein thrombosis: a retrospective cohort study

**DOI:** 10.1186/s13018-022-03457-y

**Published:** 2022-12-20

**Authors:** Xiaojuan Xiong, Shenglian Xu, Ting Li, Bo Cheng

**Affiliations:** 1 Department of Anesthesiology, Army Medical Center of PLA, Daping Hospital, Army Medical University, 10 ChangjiangZhilu, Yuzhong District, Chongqing, 400042 China; 2 Department of Anesthesiology, The First Affiliated Hospital of Chongqing Medical University, 1 Youyi Road, Yuzhong District, Chongqing, 400000 China

**Keywords:** Total joint arthroplasty, Deep vein thrombosis, Preoperative, Severity of anemia, Blood transfusion

## Abstract

**Background:**

To explore the correlation of the severity of preoperative anemia with deep vein thrombosis (DVT) in patients undergoing total joint arthroplasty (TJA).

**Methods:**

A total of 2461 TJA patients were classified into anemia and non-anemia groups or DVT and non-DVT groups. A logistic regression model was established using propensity score matching (PSM) analysis with preoperative anemia of TJA patients as a dependent variable and DVT-related variables as covariates. The caliper value was set as 0.01, and the anemia and non-anemia groups were matched based on the ratio of 1:1 (835 pairs). Finally, data of all patients were analyzed by binary logistic regression.

**Results:**

Preoperative anemia was observed in 872 cases (35.43%) and DVT in 170 cases (6.91%). Binary logistic regression after PSM revealed that the DVT risk of patients with preoperative, moderate and severe anemia increased by 1.82 [*P* = 0.00, 95% confidence interval (95% CI) (1.32–2.48)], 2.77 [*P* = 0.00, 95% CI (1.72–4.45)], and 8.26 [*P* = 0.00, 95% CI (3.22–21.16)] times, respectively. The risks of blood transfusion in the perioperative period in patients with anemia, mild anemia, moderate anemia, and severe anemia increased by 3.52 times [*P* = 0.00, 95% CI (2.78–4.47)], 2.13 [*P* = 0.00, 95% CI (1.63–2.79)], 7.22 [*P* = 0.00, 95% CI (5.30–9.83)], and 61.37 [*P* = 0.00, 95% CI (14.21–265.04)] times, respectively.

**Conclusion:**

Preoperative anemia is an independent risk factor for preoperative DVT and blood transfusion in the perioperative period for TJA patients. The more severe the preoperative anemia, the greater the risk of preoperative DVT and perioperative blood transfusion in TJA patients. Therefore, patients with preoperative anemia, especially with moderate and severe anemia, should be screened for DVT formation before undergoing TJA.

*Trial registration* ChiCRT2100054844.

## Introduction

Total joint arthroplasty (TJA) includes total hip and total knee arthroplasty (THA and TKA), and it is anticipated that the number of THA and TKA procedures will increase in the next decade [1]. Osteoarthritis (OA) of the hip and knee is frequently treated with THA and TKA, and patients have a good prognosis [2]. THA and TKA are at a high risk of developing venous thromboembolism (VTE). About 40–60% of patients undergoing hip and knee surgery experience DVT, and 4–10% develop pulmonary embolism (PE) without prevention [3, 4]. Among them, the prevalence of DVT before TKA was 6.85–17.9% [5, 6] and THA reached as high as 29.4% [7]. According to Song et al. [7], 66.7% of patients diagnosed with VTE following THA had the same preoperative thrombosis site. According to Smith et al. [8], PE can result from small thrombus loss and peripheral DVT of small volume in THA. Therefore, it is crucial to identify high-risk factors for preoperative DVT patients undergoing TJA for the perioperative prevention and treatment of DVT.

Anemia is prevalent in patients undergoing TJA; at the time of admission, 44% of them are anemic, which increases to 87% post-surgery [9]. After primary and revision TJA, increased complications and mortality are associated with preoperative anemia [10]. A significant risk of postoperative DVT, sepsis, wound infection, and wound diaphysis is seen in patients with severe anemia before TKA [11]. Preoperative anemia has also been shown to be a risk factor for increased economic burden after TJA due to higher transfusion rates, longer hospital stays, and transfusion-related complications [12]. Preoperative anemia predisposes systemic and surgical site complications during TJA [13]. Patients with moderate-to-severe anemia are more likely to experience postoperative complications than those with mild anemia, with a significant correlation between increased anemia severity and increased postoperative complications in TJA [13]. According to Cheung et al. [14], anemia was an independent risk factor for preoperative DVT in patients with hip fractures.

Presently, most studies focus on the effects of anemia before TJA on postoperative quality of life. However, there is no study to verify the connection between the level of anemia in patients before TJA and the development of preoperative DVT.

## Materials and methods

### Inclusion and exclusion criteria

Inclusion criteria: all patients who underwent TJA in our hospital between January 1, 2017, and December 31, 2021.

Exclusion criteria: patients without preoperative deep vein ultrasound of the lower limbs and a routine blood test; patients with TJA diagnosed with trauma, tumor, and tuberculosis; and patients < 20 years old.

### Research method

Our hospital treated 2772 patients who needed TJA between January 1, 2017, and December 31, 2021, and 2461 additional patients were enrolled. According to the 2011 WHO standard [15] (and the reference index of our laboratory), male hemoglobin (Hb) < 130 g/L and female Hb < 120 g/L are defined as anemic. Mild anemia, male: 110 g/L ≤ Hb < 130 g/L and female: 110 g/L ≤ Hb < 120 g/L; moderate anemia: 80 g/L ≤ Hb < 110 g/L; and severe anemia Hb < 80 g/L. Patients were divided into the anemia group and the non-anemia group based on preoperative Hb results. Patients were divided into DVT and non-DVT groups based on preoperative deep vein color ultrasound screening results to analyze the connection between preoperative anemia of TJA and preoperative DVT formation. This study has been approved by the Army Medical Center of PLA (ratification number is 2021(288)) on December 31, 2021, and registered in the WHO International Clinical Trial Registration (ChiCRT2100054844).

### Data collection

The surgical anesthesia system queries patients’ clinical data through the electronic medical record system. Two experienced ultrasound physicians performed pulsed-Doppler ultrasonography on both lower limbs of each patient using the Philips IE33 GE Vivid 9, C5-1 linear probe, and a frequency of 5–10 Hz. Venous incompressibility, intracavity filling defects, and the absence of a Doppler signal were the positive indicators for DVT. The thrombus that developed far from the popliteal vein was identified as the distal thrombus when the DVT formation site was examined. Popliteal vein and popliteal vein were proximal thrombi. A mixed thrombus is a thrombus that contains both proximal and distal thromboses. We collected basic information about the patients: patient's name, hospitalization number, height, weight, body mass index (BMI), age, sex, and preoperative diagnosis; medical records: preoperative complications including hypertension, diabetes mellitus (DM), coronary heart disease (CHD), chronic obstructive pulmonary disease (COPD), chronic bronchitis, rheumatoid arthritis (RA), OA, cerebral infarction, cancer, renal failure, use of corticosteroids, preoperative smoking, alcohol consumption, and history of major surgery within 12 months; and laboratory and auxiliary examinations: ABO blood group (type A, B, AB, and O), blood routine, Hb, red blood cell (RBC), hematocrit (HCT), platelet count (PLT), preoperative deep vein ultrasound results of the lower limbs, and blood transfusion during surgery.

### Statistical methods

The statistical analysis was performed using the statistical package for social sciences v26.0. Counting data were analyzed using the chi-square test or Fisher’s exact probability method, and the results were expressed as percentages (%) to analyze DVT correlation covariates. Preoperative TJA anemia was used as the dependent variable and DVT-related variables as the covariable in a logistic model that was created using PSM to reduce the influence of confounders. The anemia and non-anemia groups were matched in a ratio of 1:1 using 0.01 as the caliper value (20% of the standard deviation of the propensity index for both groups). A standardized difference method was used to assess the matching effect of PSM, and the standardized difference (*d*) was calculated. The matching effect is thought to be effective if *d* < 0.1. After data from the two groups were matched, binary logistic regression analysis was performed on the data to determine the correlation between anemia and the severity of anemia before TJA and DVT. The adjusted odds ratio (OR) and 95% CI were calculated as a result. *P* < 0.05 was considered statistically significant.

## Results

### Basic preoperative information of patients with TJA

**Fig. 1:**
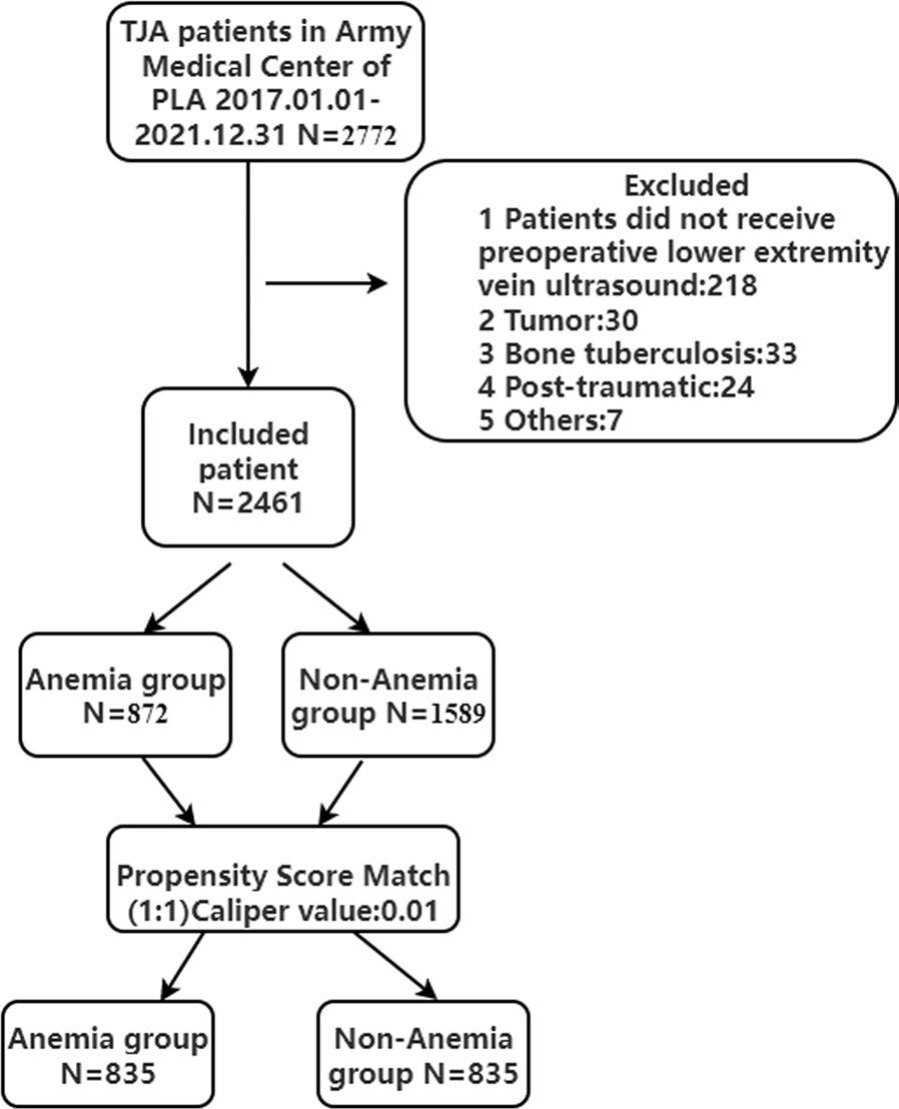
Research scheme of propensity score matching analysis of preoperative anemia and DVT in TJA patients. *TJA* total joint arthroplasty; *DVT* deep vein thrombosis

In total, 2461 (88.78%) patients were enrolled, with a sample loss of < 20% (Fig. 1). There were 1456 patients with THA and 1005 patients with TKA. All patients with TJA had routine blood tests done 24 h before the preoperative deep vein ultrasound. The average age of patients with TJA was 63.47 ± 11.75 year, with heights of 158.19 ± 8.17 cm, weights of 61.19 ± 10.41 kg, and BMI: of 24.47 ± 4.2 kg/m^2^ (Table 1). There were 872 males (35.43%) and 1589 females (64.57%). There were 872 (35.43%) patients with TJA with anemia, 568 (23.08%) perioperative blood transfusions, and 436 (26.11%) PSM-matched blood transfusions. Hypertension (701 cases, 8.5%) had the highest incidence of preoperative complications in patients with TJA, followed by DM (256 cases, 10.4%) and CHD (133 cases, 4%).

**Table 1:** Summary of patient characteristics

	Anemia (*N* = 872)	Non-anemia (*N* = 1588)	*P* value
Age (year)	66.04 ± 10.94	62.07 ± 10.94	0.00
Height (cm)	156.95 ± 7.99	158.87 ± 8.2	0.00
Weight (kg)	58.14 ± 9.72	62.86 ± 10.4	0.00
BMI (kg/m^2^)	23.69 ± 4.97	24.9 ± 3.64	0.00
Hb (g/L)	110.57 ± 12.41	135.84 ± 11.28	0.00
HCT (%)	34.27 ± 3.48	40.99 ± 3.45	0.00
PLT (10^9^/L)	216.35 ± 89.34	209.59 ± 65.43	0.05

*BMI* body mass index; *Hb* hemoglobin; *HCT* hematocrit; *P* < 0.05 was considered statistically significant

### DVT-related variables were compared between the two groups before and after PSM

Before PSM matching, there was no statistically significant difference (*P* > 0.05) between the anemia and non-anemia groups for gender, hypertension, CHD, COPD, chronic contraception, major surgery in the last 12 months, depression, and cancer. There were statistically significant differences between the anemia and the non-anemia groups in terms of diagnosis (*P* = 0.047), DM (*P* = 0.032), cerebral infarction (*P* = 0.014), corticosteroid (*P* = 0.000), smoking (*P* = 0.000), drinking (*P* = 0.000), blood type (*P* = 0.029), and renal failure (*P* = 0.000).

**Fig. 2:**
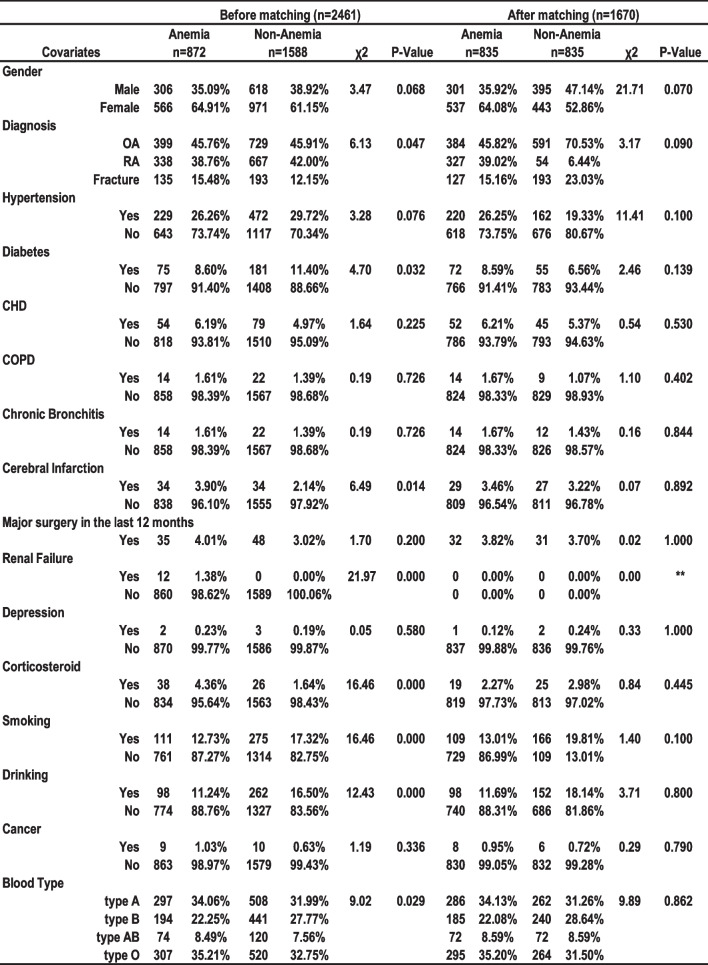
Distribution characteristics of covariates in TJA patients before and after PSM in the anemia and non-anemia groups. *CHD* coronary heart disease; *COPD* chronic obstructive pulmonary disease; *PSM* propensity score matching; *P* < 0.05 was considered statistically significant

Following PSM matching, 835 pairs of data were successfully matched, and the *P* values of all 16 variables were > 0.05, indicating no statistically significant difference between groups. The two data sets are fairly balanced and comparable (Fig. 2).

### Equilibrium after PSM matching

**Fig. 3:**
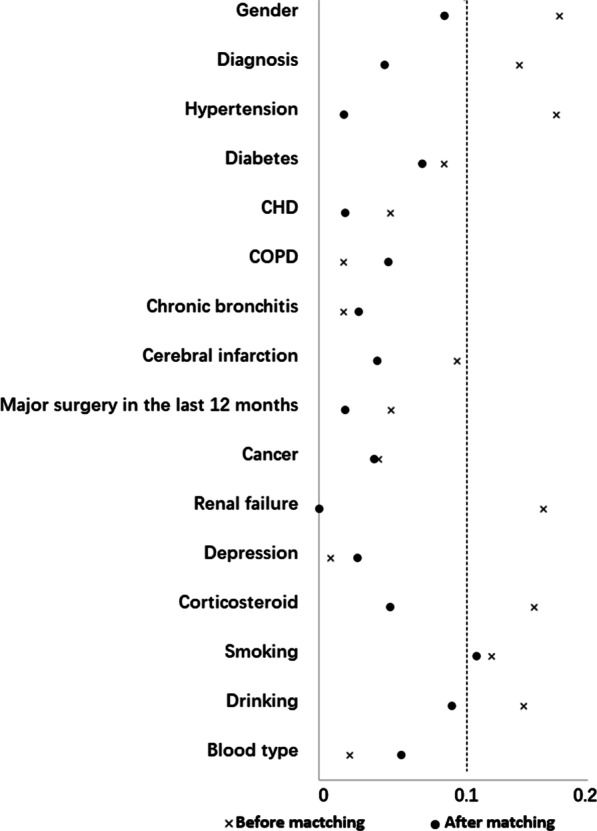
Variable standardization difference diagram. *CHD* coronary heart disease; *COPD* chronic obstructive pulmonary disease

The standardized difference between the two groups was calculated (*d*) before and after matching each covariable. Before PSM matching, there were 16 groups of data, including gender, diagnosis, hypertension, renal failure, corticosteroid, and smoking. The drinking group's *d* value was > 0.1. After PSM matching, the smoking groups' *d* value was 0.117, while the other 15 groups’ *d* value was < 0.1, indicating a successful PSM matching (Fig. 3).

### Correlation between the severity of preoperative anemia in patients with TJA and DVT

Preoperative thrombosis was observed in 2461 (170) patients with TJA, with an incidence of 6.91%. In the anemia group, there were 872 (83) cases of DVT, with an incidence of 9.52%, and there were 1589 (87) cases in the non-anemia group, with an incidence of 5.48%. Following PSM matching, there were 1670 (118) cases of DVT, representing an incidence of 7.07%. The incidence of DVT in the anemia group, 835 (76) cases, was 9.1%, and in the non-anemia group, 835 (42) cases, was 5.03%. Distal thrombosis was 73.53% in 125 cases, proximal thrombus was 11.76% in 20 cases, and mixed thrombosis was 14.71% in 25 cases. After PSM matching, 1670 cases (118) of DVT were formed with an incidence of 7.07%. Table 2, before and after PSM matching, displays the preoperative anemia level of patients with TJA and the incidence of DVT.

**Table 2:** Incidence of DVT in anemia and non-anemia groups before and after PSM matching

DVT	Non-anemia	Severity of anemia			Total	
		Mild	Moderate	Severe		
Before PSM	1502 (87)	562 (37)	284 (39)	26 (7)	2461 (170)	6.91%
After PSM	835 (42)	546 (35)	266 (34)	23 (7)	1670 (118)	7.07%

*PSM* propensity score matching; *DVT* deep vein thrombosis

According to this study, patients with anemia before TJA had a 1.89-fold higher risk of DVT before PSM matching [*P* = 0.001 95% CI (1.28–2.79)], and patients with moderate anemia had a 2.75-fold higher risk of DVT before PSM matching [*P* = 0.001 95% CI (1.84–4.10)]. Patients with severe anemia had a 6.36-fold higher risk of DVT [*P* = 0.00, 95% CI (2.60–15.54)].

**Fig. 4:**
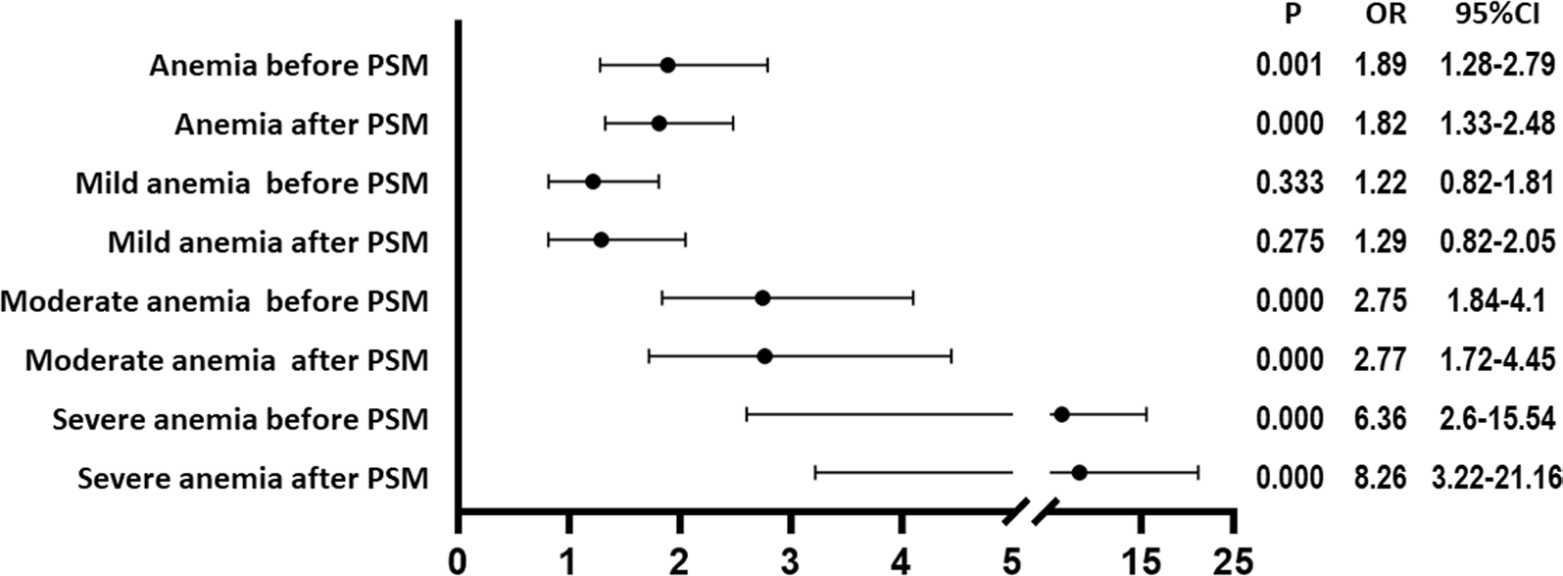
Binary logistic regression analysis of preoperative anemia severity and DVT in TJA patients. *PSM* propensity score matching; *DVT* deep vein thrombosis; *TJA* total joint arthroplasty; *CI* confidence interval; *OR* odds ratio; *P* < 0.05 was considered statistically significant

Binary logistic regression revealed that patients with anemia before TJA had a 1.82-fold higher risk of DVT after PSM matching [*P* = 0.00, 95% CI (1.32–2.48)]. Patients with moderate anemia had a 2.77-fold higher risk of DVT [*P* = 0.00, 95% CI (1.72–4.45)]. Patients with severe anemia had an 8.26-fold higher risk of DVT [*P* = 0.00, 95% CI (3.22–21.16)] (Fig. 4). However, it was not discovered that patients with mild anemia before and after TJA had a higher risk of DVT before and after PSM matching.

### Relationship between preoperative anemia severity and perioperative blood transfusion in patients with TJA

According to this study, patients with anemia before TJA had a 3.60-fold higher risk of perioperative transfusion before PSM matching [*P* = 0.00, 95% CI (2.96–4.37)]. Patients with mild anemia had a 2.15-fold higher risk of perioperative transfusion [*P* = 0.00, 95% CI (1.70–2.70)]. Patients with moderate anemia had a 7.26-fold higher risk of perioperative transfusion [*P* = 0.00, 95% CI (5.53–9.53)]. Patients with severe anemia had a 69.49-fold higher risk of perioperative transfusion [*P* = 0.00, 95% CI (16.31–295.98)].

**Fig. 5:**
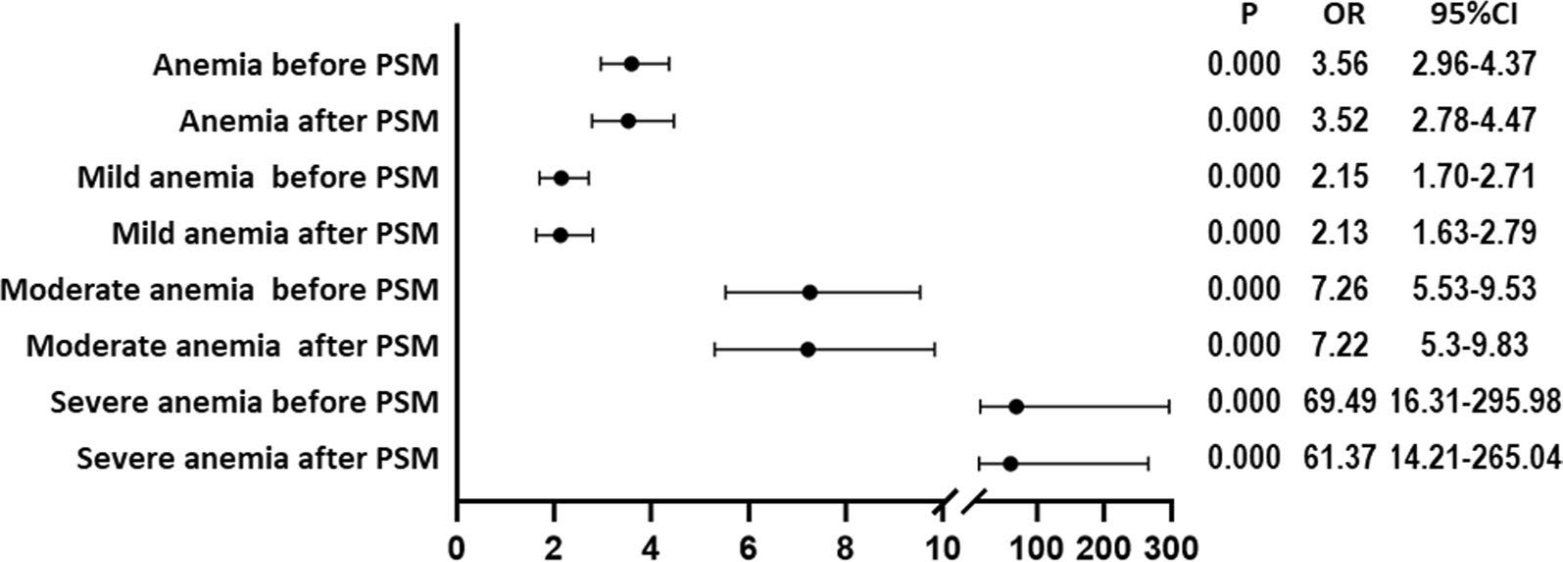
Binary logistic regression analysis of preoperative anemia severity and perioperative blood transfusion in TJA patients. *PSM* propensity score matching; *TJA* total joint arthroplasty; *CI* confidence interval; *OR* odds ratio; *P* < 0.05 was considered statistically significant

Patients with anemia following PSM matching had a 3.52-fold higher risk of perioperative transfusion [*P* = 0.00, 95% CI (2.7–4.47)]. Patients with mild anemia had a 2.13-fold higher risk of perioperative transfusion [*P* = 0.00, 95% CI (1.63–2.79)]. Patients with moderate anemia had a 7.22-fold higher risk of perioperative transfusion [*P* = 0.00, 95% CI (5.30–9.83)]. Patients with severe anemia had a 61.37-fold higher risk of perioperative transfusion [*P* = 0.00, 95% CI (14.21–265.04)] (Fig. 5).

## Discussion

### The relationship between preoperative anemia and DVT in TJA patients

The incidence of preoperative anemia in our TJA patients was 35.43%. This is consistent with Bierbaum et al.’s [16] finding of a 35% incidence of anemia among patients undergoing TJA. Coutinho et al. [17] identified anemia as a risk factor for cerebral venous thrombosis. Parvizi et al. [18] found that anemia was a risk factor for VTE formation after TJA. According to Xiong et al. [6], the decrease in preoperative RBC count was a high-risk factor for developing preoperative DVT before TKA. Feng et al. [19] demonstrated that preoperative anemia was an independent risk factor for VTE in elderly patients with hip fractures in China (OR: 0.144, 95% CI 0.026–0.799, *P* = 0.027). Malahias et al. [13] found that patients with moderate-to-severe anemia also had an increased risk of VTE after TKA. To our knowledge, this is the first study to find an association between anemia and preoperative DVT in TJA patients. This study showed that preoperative anemia in patients with TJA was an independent risk factor for developing preoperative DVT. Before surgery, the risk of DVT was 2.77-fold higher in patients with moderate anemia and 8.26-fold higher in those with severe anemia. The risk of preoperative DVT formation increased with the severity of preoperative anemia.

The majority of our patients were RA and OA and had chronic inflammation for a long time. Systemic inflammatory mediators were found in OA and RA: interleukin-1, interleukin-6 (IL-6), tumor necrosis factor (TNF), and interleukin-17 [20, 21]. The life span of RBCs is shortened by these cytokines, which can also inhibit RBC production [22]. Elevated IL-6 and TNF during systemic inflammatory responses are associated with an increased risk of VTE [23]. At the same time, our patients were elderly patients, with an average age of 63 years. Elderly patients undergoing THA and TKA have a 27% incidence of iron deficiency [24]. Reactive PLT hyperemia, which is frequently linked to iron deficiency, can cause hypercoagulability [25]. EPO (erythropoietin) may be increased by transient blood loss in patients with hip fractures at risk for acute blood loss. According to Goodnough et al. [26], EPO response is linearly logarithmic with changes in Hb levels, meaning that the more Hb drops, the stronger the EPO response. EPO can increase PLT and RBCs count and blood viscosity, which can both cause hypercoagulability and increase the risk of thrombosis [27, 28].

Preoperative anemia increases the risk of DVT formation before TJA. The more severe the anemia, the higher the risk of preoperative DVT in TJA patients. Therefore, TJA patients with preoperative anemia, especially moderate-to-severe anemia, should be screened for the formation of preoperative DVT.

### Relationship between the severity of preoperative anemia and blood transfusion

According to Wang et al. [29], patients with mild anemia had a 4.7-fold higher risk of postoperative blood transfusion, and those with moderate or severe shoulder arthroplasty had a 23.8-fold increased risk. These findings are consistent with the results of the present study. Our study found that preoperative anemia in patients with TJA was associated with a 3.52-fold increased risk of perioperative blood transfusion. In addition, patients undergoing TJA had a 2.13-fold higher risk of perioperative blood transfusion in patients with mild anemia before surgery, 7.22-fold higher risk in patients with moderate anemia, and 61.37-fold higher risk in patients with severe anemia. Patients with moderate or severe preoperative anemia have a higher in-hospital mortality rate, a longer hospital stay, and more intensive care when undergoing noncardiac surgery [30]. Gu et al. [13] found that the increase in preoperative anemia severity was independently associated with increased postoperative complications and mortality within 30 days in patients with primary TJA. Similarly, Grosso et al. [31] discovered that the severity of anemia after THA significantly increased postoperative complications and mortality of patients with primary TJA. Gu et al. [32] reported that patients with moderate-to-severe anemia before surgery were at greater risk of complications after TJA revision surgery than patients with mild anemia. An independent risk factor for any complications, such as specific medical complications, wound problems, septic, and bleeding complications, and mortality after TJA revision, was anemia. Sim et al. [33] discovered that patients with mild anemia before surgery had a hazard ratio (HR) of 1.98, while those with moderate and severe anemia had an HR of 2.86.

### Optimization of patients with preoperative anemia before TJA

According to this study, the risk of perioperative blood transfusion increased with the severity of preoperative anemia in patients with TJA, but there were also many adverse reactions. Although all transfusion effectively improves anemia, it may increase the risk of virus transmission, transfusion reaction, and possible immunosuppression [34]. According to Glance et al., transfusion during noncardiac surgery increases the risk of 30-day mortality and pulmonary, septic, wound, and thromboembolic complications [35]. For THA, Engoren M et al. discovered that blood transfusion was associated with a higher risk of death 90 days after surgery [36]; Koval et al. found that the blood transfusion group had a higher rate of urinary tract infections [37]; Carson et al. reported that blood transfusion increased the risk of severe bacterial infections and pneumonia [38]; Levi et al. reported a high incidence of wound infection following blood transfusion [39].

Therefore, we recommend that patients with moderate-to-severe anemia should optimize their anemia status before surgery, so as to reduce the amount of blood transfusion during the perioperative period and reduce the impact of anemia related complications. Preoperative anemia detection should be done as soon as possible, at least 14 days before elective surgery, preferably > 30 days before surgery for OA and RA patients undergoing TJA [40]. Oral and intravenous iron supplementation and EPO are strategies to improve preoperative anemia. Additionally, oral and intravenous iron supplementation is often used for mild or moderate anemia because they are practical and affordable [41]. The use of patient blood management in TJA has been shown to reduce blood transfusion, hospital stay, morbidity, and readmission [39]. Patients with severe anemia might have enough time to determine the cause and proceed with antigenic treatment. In contrast, patients with fractures are subject to a deadline operation. A low dose of EPO taken daily for 5 days before THA significantly reduced perioperative blood loss, improved postoperative Hb level, and did not increase the risk of complications compared to EPO taken 3 days before THA or the day of surgery [42]. It was suggested that starting EPO (150 IU/kg) three days before TJA is preferable to begin on the day of surgery because it is more effective in increasing Hb levels and reducing blood loss without additional complications [43]. Therefore, we advise patients with moderate-to-severe anemia to improve their condition before surgery.

The preoperative basic medical history, preoperative laboratory examination, preoperative auxiliary examination, and other data of patients with DVT and TJA were compared in this study. However, the study has some limitations. Since it is a retrospective study, some of the data are insufficient. Future studies with more sufficient data should be conducted to further verify.

## Conclusion

Preoperative anemia is an independent risk factor for preoperative DVT and blood transfusion in the perioperative period for TJA patients. The more severe the preoperative anemia, the greater the risk of preoperative DVT and perioperative blood transfusion in TJA patients. Therefore, patients with preoperative anemia, especially with moderate and severe anemia, should be screened for DVT formation before undergoing TJA.

## Data Availability

The datasets used and/or analyzed during the current study are available from the corresponding author upon reasonable request.

## Abbreviations

BMI: Body mass index
CHD: Coronary heart disease
COPD: Chronic obstructive pulmonary disease
CI: Confidence interval
DM: Diabetes mellitus
DVT: Deep vein thrombosis
EPO: Erythropoietin
Hb: Hemoglobin
HCT: Hematocrit
HR: Hazard ratio
Hs-CRP: Hypersensitive serum C-reactive protein
HR: Hazard ratio
IL-1: Interleukin-1
IL-17: Interleukin-17
IL-6: Interleukin-6
OA: Osteoarthritis
OR: Odds ratio
PE: Pulmonary embolism
PSM: Propensity score matching
RA: Rheumatoid arthritis
RBC: Red blood cell
SF: Synovial fluid
TF: Tissue factor
THA: Total hip arthroplasty
TJA: Total joint arthroplasty
TKA: Total knee arthroplasty
TNF: Tumor necrosis factor
PLT: Platelets
VTE: Venous thromboembolism
